# Clinical Effectiveness of IncobotulinumtoxinA Using a Standardized Protocol in the Management of Myogenous Temporomandibular Disorders: A 12-Month Retrospective Study

**DOI:** 10.3390/toxins18050220

**Published:** 2026-05-07

**Authors:** David Faustino Ângelo, Henrique José Cardoso, Marcella Sarkis, Kelly Santos, Francesco Maffia, David Sanz, Francisco Salvado

**Affiliations:** 1 Instituto Português da Face, 1500-493 Lisbon, Portugal; marcella.sarkis@ipface.pt (M.S.); kelly.santos@ipface.pt (K.S.); francesco.maffia@ipface.pt (F.M.); david.sanz@ipface.pt (D.S.); 2 Centre for Rapid and Sustainable Product Development, Polytechnic Institute of Leiria, 2430-028 Marinha Grande, Portugal; 3 Faculty of Medicine, Lisboa University, 1649-028 Lisbon, Portugal; 4 Serviço de Estomatologia, Hospital de Egas Moniz, Centro Hospitalar de Lisboa Ocidental, 1349-019 Lisbon, Portugal; 5 Clinica Universitária de Estomatologia, Centro Hospitalar Universitário Lisboa Norte (CHUNL), 1648-028 Lisbon, Portugal; fjsalvado@medicina.ulisboa.pt

**Keywords:** botulinum toxin type A, incobotulinumtoxinA, masticatory muscles, myofascial pain, orofacial pain, temporomandibular joint, temporomandibular disorders

## Abstract

Background: Myogenous temporomandibular disorders (TMDs) are a common subtype of orofacial pain. Evidence regarding treatment with botulinum toxin type A (BoNT-A) remains heterogeneous, and its use is generally limited to refractory cases. This study evaluated 12-month clinical outcomes following an incobotulinumtoxinA protocol (the Ângelo Botulinum Toxin Protocol^®^) in adults with DC/TMD-confirmed myogenous TMD unresponsive to conservative therapy. Methods: This retrospective observational study reviewed records from 98 adults treated with incobotulinumtoxinA following the predefined injection protocol. All patients had failed ≥ 3 months of conservative management and completed ≥ 12 months of follow-up. Outcomes included myalgia severity (0–3), patient-reported orofacial pain intensity (VAS 0–10), and maximum mouth opening (MMO). Favorable outcome criteria required myalgia 0–1 or VAS ≤ 2 and MMO ≥ 35 mm. Results: Myalgia significantly decreased at 12 months (2.69 ± 0.64 to 0.43 ± 0.85; *p* < 0.001). Patient-reported orofacial pain intensity also improved (2.44 ± 2.54 to 0.37 ± 1.33; *p* < 0.001). MMO remained stable, indicating preserved mandibular mobility. Overall, 79.6% of patients met the predefined favorable outcome criteria. Reintervention was required in 12 patients; 7 received additional incobotulinumtoxinA injections, and 5 underwent TMJ arthrocentesis. No complications were observed. Conclusion: This protocol was associated with improvements in muscular pain and orofacial discomfort while preserving mandibular mobility. However, given the retrospective design and absence of a control group, these findings should be interpreted as hypothesis generating.

## 1. Introduction

Temporomandibular disorders (TMDs) comprise a heterogeneous group of musculoskeletal conditions characterized by dysfunction of the masticatory muscles, the temporomandibular joint (TMJ) and associated structures involved in mandibular movement [1]. Pain, TMJ dysfunction, reduced jaw mobility and limitations in basic oral activities are manifestations of these conditions, which may affect quality of life [2,3]. Reported prevalence rates of TMD in the general population range from 29.5% to 34% [4,5] and are expected to increase to 44% by 2050, with females being more susceptible than males [5,6].

In the majority of cases, TMDs may originate in the masticatory muscles or in the temporomandibular joint, referred to as myogenous and arthrogenous TMDs, respectively, and may also present with a mixed origin [7]. Myogenous TMDs are the most common type of TMD, representing 45.3% of TMDs [8,9]. The etiopathogenesis of myogenous TMDs is multifactorial, including trauma, parafunctional behaviors, central sensitization, inflammation, and psychological factors, which render the diagnosis and management of these disorders particularly challenging [7,10,11,12].

Given the limited understanding of the etiopathogenesis of these disorders, there is no universally accepted standard treatment, and existing approaches are generally grouped into two main categories: (1) Conservative therapies, which include patient education, pharmacological agents such as non-steroidal anti-inflammatory drugs (NSAIDs), occlusal splint, physiotherapy, electrical therapy and psychological intervention; and (2) minimally invasive treatments, including dry needling, acupuncture and injections [9]. Among the different drugs used for minimally invasive injections, botulinum toxin type A (BoNT-A) offers potential therapeutic benefits and is increasingly studied for myogenous TMD [13].

BoNT-A is a neurotoxin produced by Clostridium botulinum that inhibits acetylcholine release at neuromuscular junctions by binding to presynaptic cholinergic and nerve terminals, which results in muscle relaxation and reduced pain [14]. At the peripheral level, inhibitory actions on pain receptors and pain transmitters can reduce peripheral sensitization, partly by blocking the release of neurotransmitters involved in pain pathways such as substance P, calcitonin gene-related peptide (CGRP), and glutamate [14]. BoNT-A may also interfere with inflammatory cascades, for example, by decreasing pro-inflammatory cytokines following intra-articular administration [15], and may act at sensory ganglia by attenuating pain-induced upregulation of ion channels implicated in nociception, including TRPV1 [16]. In addition, a central analgesic component has been proposed, involving reductions in central sensitization mediated by changes in neurotransmitter release, modulation of microglial activation, and synergistic effects on opioid and GABAergic systems [15,17]. BoNT-A has been evaluated for its use as a treatment for myogenous TMD in multiple clinical trials. Despite growing evidence supporting its analgesic effects, substantial heterogeneity persists regarding clinical outcomes, dosing strategies, injection sites, and long-term therapeutic stability, which limits the establishment of standardized treatment protocols. An umbrella review that collects systematic reviews assessing BoNT-A treatment effects on pain intensity, mandibular movements, and adverse events in patients with myogenous TMD was published in 2024 [18]. Even though there is evidence suggesting potential effectiveness of BoNT-A in reducing pain intensity, there are still differences between studies regarding pain reduction and mandibular movements [18]. Another key point of BoNT-A treatments is their administration, with existing different formulations and sites of injection [19]. The most common injection sites are the masseter and temporalis muscles, followed by the pterygoid muscle, with unilateral or bilateral injections depending on the study [13,20,21].

Therefore, the objective of this study was to describe 12-month clinical outcomes associated with incobotulinumtoxinA administration using a standardized protocol, in patients with Diagnostic Criteria for Temporomandibular Disorders (DC/TMD)-confirmed myogenous TMD resistant to conservative therapy. Clinical outcomes included changes in myalgia severity, patient-reported orofacial pain intensity, mandibular mobility, and the emergence of arthrogenous signs during follow-up.

## 2. Results

**Figure 1:**
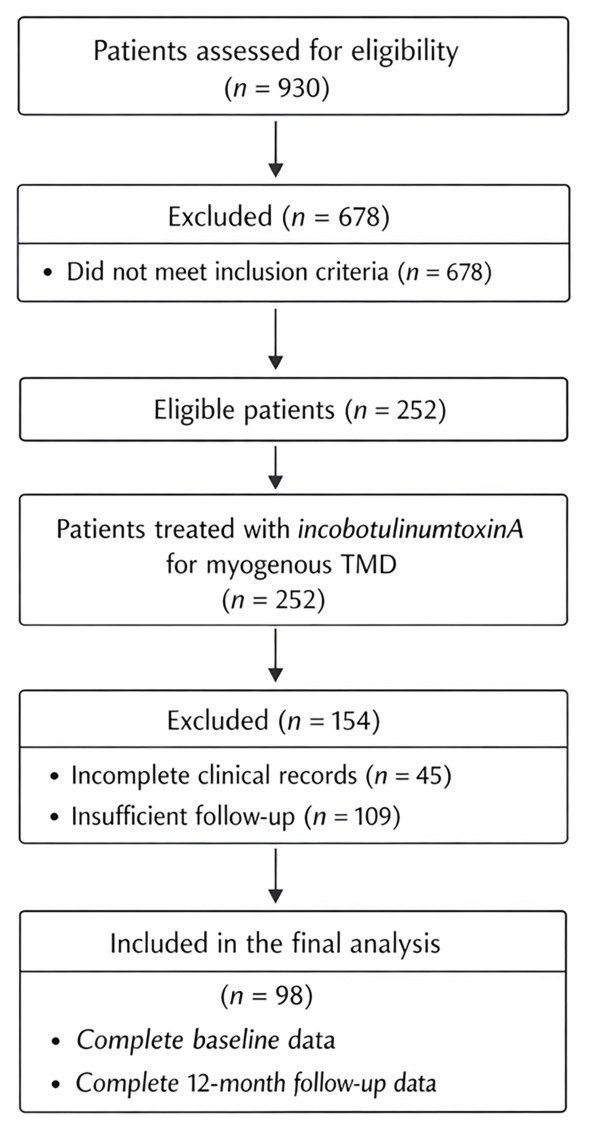
Flow diagram of patient selection for inclusion in the retrospective study of incobotulinumtoxinA treatment for myogenous temporomandibular disorder.

A total of 930 patients were assessed for eligibility during the study period. Of these, 678 were excluded for not meeting the inclusion criteria. The remaining 252 patients underwent treatment with incobotulinumtoxinA for myogenous TMD. Among them, 154 patients were further excluded due to incomplete clinical records (*n* = 45) or insufficient follow-up (*n* = 109). Ultimately, 98 patients with complete baseline and 12-month follow-up data were included in the final analysis (Figure 1).

The mean age was 45.55 ± 16.27 years (range: 18–77), and the majority were female (85.71%, *n* = 84) (Table 1).

All patients completed a minimum of 12 months of follow-up, with a mean duration of 401.13 ± 66.20 days (range: 309–490). At baseline, all patients presented with a clinical diagnosis of myogenous TMD. The median myalgia severity score at baseline was 3.0 (IQR: 2.0–3.0) on the 0–3 scale, indicating predominantly moderate to severe muscular pain. All patients were diagnosed exclusively with myogenous TMD at baseline. At baseline, patient-reported orofacial pain intensity had a mean VAS of 2.44 ± 2.54. Mean maximum mouth opening (MMO) at baseline was 42.55 ± 16.72 mm, reflecting variability in mandibular function across patients (Table 2).

**Figure 2:**
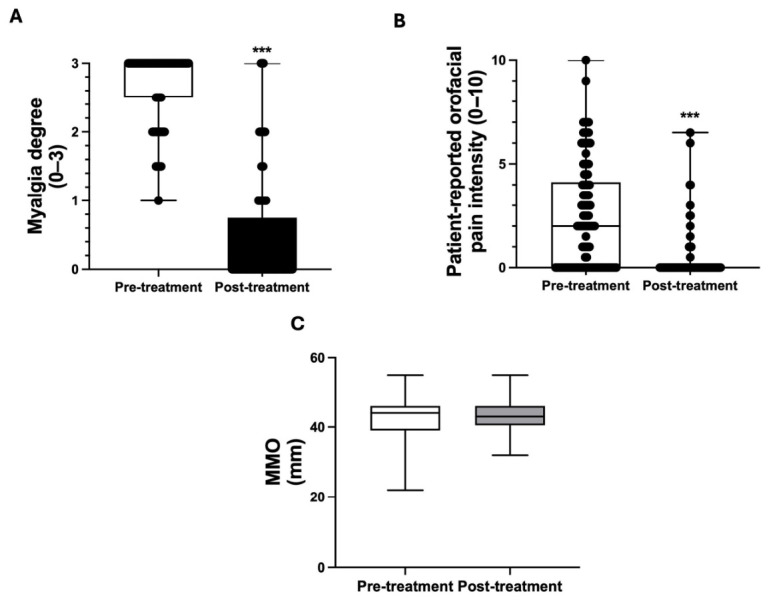
Graphical representation of the three main clinical variables assessed pre- and post-treatment. (**A**) Myalgia severity (ordinal scale 0–3), presented as boxplots with median, interquartile range, and individual data points, demonstrating a significant reduction over time. (**B**) Patient-reported orofacial pain intensity measured using a visual analogue scale (VAS, 0–10), showing a significant decrease between time points. (**C**) Maximum mouth opening (MMO, mm), showing no significant change between baseline and follow-up. *** *p* < 0.001.

At the 12-month follow-up, a significant reduction in myalgia severity was noted compared to baseline values (3.0 (IQR: 2.0–3.0) vs. 0.0 (IQR: 0.0–0.0), *p* < 0.001, Figure 2A), with a large effect size (r = 0.86). Most patients reported mild or no muscular pain at final evaluation. Patient-reported orofacial pain intensity also showed significant improvement, with VAS scores decreasing from 2.44 ± 2.54 at baseline to 0.37 ± 1.15 at 12 months (mean difference: −2.07; 95% CI: −2.62 to −1.52; *p* < 0.001, Figure 2B), with a moderate-to-large effect size (Cohen’s d = 0.76).

Maximum mouth opening (MMO) remained stable throughout the follow-up period, with no statistically significant differences observed between baseline and 12-month measurements, indicating preserved functional mandibular range (mean difference: 0.55 mm; 95% CI: −0.61 to 1.71; *p* = 0.35, Figure 2C), with a negligible effect size (Cohen’s d = 0.10).

The individual components of the composite outcome are presented in Table 3. At baseline, a high proportion of patients already exhibited functionally adequate MMO values (96.9%), while favorable levels of patient-reported pain intensity (VAS ≤ 2) were observed in 56.1% of cases, and no patients met the predefined criteria for low myalgia severity (grade 0–1). At 12-month follow-up, substantial improvements were identified in pain-related outcomes, with 84.7% of patients achieving low myalgia severity and 92.9% reporting VAS ≤ 2. In contrast, MMO showed minimal change over time, with 99.0% of patients maintaining values ≥ 35 mm.

Based on the predefined favorable outcome criteria, 79.6% of patients (*n* = 78) achieved a favorable outcome at 12 months. Among the 20 patients (20.4%) classified as treatment failures, 12 (12.2%) required reintervention during follow-up. Of these, 7 patients received repeat incobotulinumtoxinA injections, while 5 underwent TMJ arthrocentesis (Table 4). During follow-up, a minority of patients presented clinical findings suggestive of arthrogenous involvement (*n* = 5, 5.1%).

No major adverse events were documented in the available clinical records; however, adverse events were not systematically recorded. Reported adverse effects were limited to transient local discomfort and mild muscle fatigue, as documented in clinical records.

## 3. Discussion

TMDs constitute a complex and heterogeneous group of musculoskeletal conditions affecting the masticatory muscles and TMJ. This condition is considered a major global public health concern, as it represents the most common form of chronic orofacial pain and considerably decreases patients’ quality of life [9]. Since the underlying causes of TMDs are complex and not fully understood, no universal treatment exists. Instead, a variety of therapeutic approaches have been proposed over time [7].

The results suggest improvements in muscular pain and TMJ-related discomfort, with preservation of mandibular mobility, positioning this study within the expanding—yet heterogeneous—body of literature supporting the use of BoNT-A in myogenous TMD, particularly in patients who do not respond to conservative approaches [22,23]. Importantly, all patients in this study met strict Diagnostic Criteria for Temporomandibular Disorders (DC/TMD), enhancing diagnostic precision and internal validity. This aligns with current recommendations suggesting that BoNT-A should be considered after failure of conventional therapies due to its risk–benefit profile [18]. In this regard, the umbrella review by De La Torre Canales et al. (2024) concluded that BoNT-A demonstrates effectiveness in reducing pain intensity; however, the overall quality of evidence remains inconsistent, and adverse event considerations support reserving this intervention for refractory cases [18].

Within this framework suggesting a potential role for predefined administration approaches for myogenous TMD refractory patients, a marked reduction in myalgia was noted at 12 months, with the mean myalgia degree decreasing from a predominantly moderate–severe level at baseline to a near-absence of pain in most patients. Similar improvements were noted in patient-reported orofacial pain intensity. Pain perception in TMD is complex, and patients may have difficulty distinguishing between muscular and joint-related pain, particularly in the orofacial region where symptoms frequently overlap. Furthermore, as this cohort included exclusively patients with myogenous TMD, lower baseline pain intensity compared to arthrogenous or mixed TMD presentations may be expected. These factors should be considered when interpreting the magnitude of clinical improvement observed. These findings are consistent with evidence from randomized trials and systematic reviews demonstrating clinically meaningful, although variable, analgesic effects of BoNT-A compared to placebo. In the study by De la Torre Canales et al. [24], a ≥ 30% pain reduction was reported by 20/20, 17/20, and 17/20 patients treated with 40U, 70U, and 100U of BoNT-A, respectively, in the six-month follow-up, whereas only 7/20 placebo patients reached this threshold. Subsequent findings demonstrated dose-dependent improvements in psychosocial and pain-related outcomes [25]. Comparable results were reported in other randomized trials, comparing BoNT-A to saline or acupuncture [26]. Additionally, a retrospective study demonstrated a mean 50% reduction in pain after four months of BoNT-A therapy [27]. Conversely, some studies found no superiority of BoNT-A over placebo regarding pain reduction, reinforcing the heterogeneity noted in the evidence base and highlighting the influence of diagnostic criteria, dosing protocols and patient selection [28,29,30]. Meta-analytic evidence indicates that BoNT-A reduces pain intensity to a greater extent than saline placebo in the short and medium term, despite high heterogeneity across study designs, diagnostic criteria, and dosing strategies [13].

The observed clinical improvement in this cohort may be explained by the multifactorial mechanisms of action of BoNT-A. Beyond neuromuscular blockade through inhibition of acetylcholine release, BoNT-A modulates neurogenic inflammation and reduces the release of neurotransmitters and neuropeptides involved in pain transmission, including substance P and calcitonin gene-related peptide (CGRP). Additionally, central mechanisms involving modulation of microglial activation and neurotransmitter pathways have been proposed [31]. These non-motor effects provide a biological rationale for the durable reduction in muscular pain observed in this study and are consistent with broader evidence regarding BoNT-A in chronic pain management [15,32,33].

In addition to symptom reduction, MMO remained stable throughout follow-up, indicating preservation of mandibular mobility. This finding may be clinically relevant, as concerns have been raised that excessive or repeated BoNT-A dosing could impair muscle function or reduce mechanical capacity [34]. The stability of MMO in this cohort suggests that the standardized dosing strategy applied may mitigate functional compromise, although imaging-based investigations would be required to definitively exclude subclinical structural changes.

Approximately 80% of patients achieved a favorable outcome at one year, a proportion comparable to other retrospective cohorts evaluating botulinum toxin in myogenous TMD [27]. The reported favorable outcome rate should be approached with caution, as the analysis of its individual outcomes demonstrated that mandibular function was largely preserved at baseline, and that the noted clinical benefit was predominantly driven by improvements in myalgia severity and patient-reported pain intensity. Consequently, the favorable outcome criteria reported in this study appear to be primarily influenced by pain-related outcomes, while MMO mainly served to confirm the absence of functional deterioration rather than to contribute meaningfully to treatment success. Among the minority classified as treatment failures, some required reintervention either with repeat incobotulinumtoxinA injections or TMJ arthrocentesis. Notably, a small proportion of patients developed signs suggestive of arthrogenous involvement during follow-up. Given that the presence of arthrogenous TMD was an exclusion criterion at baseline, these findings likely reflect the multifactorial and dynamic nature of TMDs, in which behavioral factors, persistent parafunctional habits, and biomechanical overload may contribute to disease progression over time. Importantly, the relatively low proportion of such cases observed in this cohort suggests that this progression is unlikely to be attributable to the botulinum toxin intervention itself.

Furthermore, the integration of physiotherapy and behavioral counseling following BoNT-A injections represents an important component of the therapeutic strategy. Evidence increasingly supports multimodal approaches to myogenous TMD, reflecting the complex interplay of biomechanical, inflammatory and psychosocial factors [9]. Although the present design does not allow isolation of the specific contribution of adjunctive therapies, their inclusion mirrors contemporary clinical practice and may have enhanced the durability of symptom improvement. Therefore, the observed outcomes should be interpreted as the result of a multimodal therapeutic approach rather than the isolated effect of incobotulinumtoxinA.

Safety considerations remain critical. Adverse events were not systematically recorded, which limits the ability to draw definitive conclusions regarding safety. Therefore, the safety profile observed in this study should be interpreted with caution. Previous studies have described dose-dependent reductions in masticatory muscle thickness and mandibular bone density with repeated BoNT-A exposure [21,34]. Conversely, recent meta-analyses have reported no significant differences in adverse event rates between BoNT-A and placebo [13,20]. The BoNT-A formulation used in this study (incobotulinumtoxinA, Xeomin^®^) is the only pure neurotoxin (150 kDa) and has demonstrated significantly lower immunogenicity compared to other available toxins [35,36] with no reported cases of loss of response due to immune resistance [37]. Considering that one of the main reasons for discontinuation in patients treated with botulinum toxin is the lack of benefit or reduced response to treatment [38], often associated with the development of immunogenicity to botulinum toxin [39], using incobotulinumtoxinA from the start is recommended to minimize the risk of reduced treatment response and ensure optimal long-term outcomes [40]. Switching patients promptly to a less immunogenic botulinum toxin is recommended, as neutralizing antibodies may develop before clinical resistance becomes evident [36].

Beyond the immediate clinical outcomes, the present findings may contribute to an emerging therapeutic rationale centered on TMJ unloading through neuromuscular modulation [41]. These findings may support the concept of Preoperative Unload Joint with Botulinum Toxin (PUB-TT), based on the idea that reduction of masticatory muscle hyperactivity can contribute to decreased joint loading. Future prospective investigations are warranted to explore the potential role of BoNT-A-mediated joint decompression as a preparatory step before invasive or surgical temporomandibular procedures. However, this concept remains exploratory and requires dedicated investigation in future studies.

This study also carries methodological strengths. The strict application of DC/TMD criteria enhances diagnostic precision, addressing a long-standing limitation in BoNT-A research where inconsistent diagnostic standards have hindered comparability across studies. The 12-month follow-up represents one of the longest real-world evaluations of BoNT-A for myogenous TMD available to date, allowing the observation of long-term treatment effects. Nevertheless, important limitations must be acknowledged. The retrospective design and absence of a control group limit causal inference. Intermediate follow-up data were not systematically available for all patients, limiting the ability to characterize the temporal evolution of clinical outcomes. The lack of systematic adverse event recording represents an important limitation of this study. Furthermore, the single-center design and highly selected refractory cohort may restrict generalizability. Future multicenter, prospective, and controlled studies are required to further clarify optimal dosing strategies, long-term structural safety, and patient selection criteria.

## 4. Conclusions

In this retrospective cohort of patients with myogenous TMD, the use of a standardized incobotulinumtoxinA injection protocol (Ângelo Botulinum Toxin Protocol^®^) was associated with improvements in muscular pain and orofacial discomfort over a 12-month period.

Given the retrospective design, absence of a control group, and use of adjunctive therapies, these findings should be interpreted with caution and considered hypothesis-generating.

Further prospective controlled studies are warranted to clarify the specific contribution of incobotulinumtoxinA and to optimize patient selection and treatment protocols.

## 5. Materials and Methods

### 5.1. Study Design

This retrospective observational study reviewed medical records of adult patients diagnosed exclusively with myogenous TMD and treated with incobotulinumtoxinA (Xeomin^®^; Merz Pharmaceuticals GmbH, Frankfurt Am Main, Germany) between 1 June 2022 and 1 June 2025. The study was conducted in accordance with the Declaration of Helsinki and approved by the Instituto Português da Face Ethics Committee (Approval No. PT/IPFace/RCT/0622/01; approved on 4 December 2023).

### 5.2. Outcomes Assessment and Evaluation

Male and female patients diagnosed with myogenous TMD were considered eligible for inclusion. The inclusion criteria were: (1) age ≥ 18 years; (2) diagnosis of myogenous TMD based on the Diagnostic Criteria for Temporomandibular Disorders (DC/TMD) criteria confirmed by clinical examination and absence of clinical or imaging signs of arthrogenous involvement; (3) failure of at least 3 months of conservative management before incobotulinumtoxinA injection; (4) minimum follow-up duration of 12 months. Exclusion criteria included: (1) history of TMJ surgery; (2) cognitive impairment interfering with pain assessment or follow-up; (3) pregnancy or breastfeeding at baseline or during follow-up; (4) presence of clinical or imaging signs of arthrogenous TMD; (5) prior facial treatment with botulinum toxin type A within the preceding 12 months; (6) diagnosis of other neuromuscular disorders or history of facial trauma.

Clinical data were retrospectively collected from medical records at the 12-month follow-up visit. Although patients attended routine clinical follow-ups at approximately 3- and 6-months visits, these data were not consistently recorded in a standardized format and were therefore not included in the formal analysis; all outcome measures were assessed based exclusively on the 12-month evaluation. Only cases with complete records of the baseline and final 12-month evaluations were included. All clinical assessments, including baseline and follow-up evaluations, were performed by the same experienced clinician using standardized examination procedures to minimize inter-examiner variability.

Treatment resistance was defined as persistence of symptoms despite at least 3 months of structured conservative management, including pharmacological therapy (e.g., non-steroidal anti-inflammatory drugs or muscle relaxants), physiotherapy, and/or occlusal splint use, without satisfactory clinical improvement as documented in clinical records. All patients were diagnosed according to the DC/TMD through a standardized clinical examination performed by an experienced clinician. This included assessment of pain location, evaluation of pain during mandibular function, and confirmation of familiar pain upon palpation of the masseter and temporalis muscles. To exclude arthrogenous involvement, clinical examination findings were complemented by imaging evaluation (MRI or CBCT) when clinically indicated, particularly in the presence of joint-related symptoms or diagnostic uncertainty.

The primary outcome was degree of myalgia, assessed by bilateral digital palpation of the masseter and temporalis muscles, applying standardized pressure (1 kg for 5 s). Pain response was graded on a four-point scale: 0 = no pain/pressure only; 1 = mild pain; 2 = moderate pain; and 3 = severe pain. Myalgia was confirmed if pain was elicited upon palpation in conjunction with a history of muscular discomfort in the jaw, preauricular region, or temples within the previous 30 days.

Secondary outcomes included patient-reported orofacial pain intensity, maximum mouth opening (MMO) and the development of clinical signs suggestive of arthrogenous TMD (e.g., articular pain, joint sounds, or functional limitation) during follow-up. Patient-reported orofacial pain intensity was assessed using a 10-point Visual Analogue Scale (VAS), where 0 indicated no pain and 10 represented the worst imaginable pain. Patients were instructed to rate their perceived pain intensity in the TMJ region and the associated masticatory muscles at rest and during function. The final score reflected the overall orofacial pain, including both muscular and joint-related components. MMO was measured as the interincisal distance between the upper and lower central incisors, recorded in millimeters using a calibrated ruler. All outcome measures were analyzed at the patient level. The reference to the number of temporomandibular joints reflects anatomical involvement rather than the unit of statistical analysis. Treatment outcomes were classified as favorable or failure based on predefined criteria combining myalgia severity, patient-reported pain intensity, and mandibular mobility (Table 5). These thresholds were selected based on their clinical relevance, with myalgia grades 0–1 reflecting minimal or absent muscular pain, VAS ≤ 2 indicating clinically favorable pain intensity, and MMO ≥ 35 mm representing functional mandibular opening, in accordance with commonly accepted clinical standards [2,42,43,44].

### 5.3. IncobotulinumtoxinA Masticatory Muscles Injection

All patients received intramuscular injections of incobotulinumtoxinA (Xeomin^®^, Merz Pharmaceuticals GmbH, Frankfurt Am Main, Germany) following an administration protocol (Ângelo Botulinum Toxin Protocol^®^), developed for the management of myogenous TMD. The protocol follows a standardized framework in terms of anatomical targets and general dose distribution, while allowing controlled clinical adaptation based on individual patient characteristics, including muscle mass, symptom distribution, and functional findings. The selection of injected muscles was based on their established role in masticatory function, parafunctional activity, and pain generation in myogenous TMD. All injections were performed by an experienced maxillofacial surgeon using an anatomical landmark-based technique, with careful palpation and, when necessary, activation of the target muscle to confirm correct localization. A systematic aspiration technique was applied prior to injection to reduce the risk of intravascular administration. Injections were performed slowly under continuous clinical monitoring. Region-specific precautions were applied for each anatomical area to avoid adjacent neurovascular structures, including facial nerve branches, major vessels, and glandular structures. Intraoral injections were performed with careful anatomical orientation to ensure accurate muscular targeting. These procedures are consistent with established safety principles for botulinum toxin administration in craniofacial musculature. A total dose of approximately 200 units (U) of incobotulinumtoxinA was used as a reference, with minor adjustments made according to individual clinical assessment. IncobotulinumtoxinA was reconstituted in sterile 0.9% saline solution. A 3 mL syringe was connected to a 21-G (0.8 mm × 40 mm) hypodermic needle and filled with exactly 2.0 mL of saline solution. The needle connected to the syringe was inserted in the 100 U vial, and a gentle dilution was performed. After disconnecting the syringe, the 21 G needle was connected to two 1.0 mL insulin syringes and filled. This was repeated with another 100 U vial, obtaining four 1.0 mL syringes with 50 U each (0.1 mL = 5 U). The syringes were connected to 30 G (0.26 × 12 mm) hypodermic needles. Skin asepsis was performed with Cutasept^®^ (BODE Chemie GmbH, Hamburg, Germany) and intra-oral asepsis with chlorhexidine solution. No anesthesia or sedation was required for the procedure. Anatomical landmarks were palpated, and the muscle was activated (when needed) to identify the optimal injection sites. The procedure was initiated on the right hemiface, in the masseter muscle. The index finger was placed parallel to the lower border of the mandible, and the first injection was performed in the anteroinferior portion of the muscle: 0.2 mL = 10 U. The second injection was performed in the posteroinferior portion of the masseter: 0.2 mL = 10 U. Lastly, a 0.1 mL corresponding to 5 U was administered at the imaginary apex of a triangle formed by the first two injections points. In the temporalis muscle, the first injection was administered into the temporalis tendon, 0.1 mL = 5 U and each of the anterior, medial, and posterior zones of the temporalis muscle received 0.2 mL = 10 U. In the neck area, the sternocleidomastoid muscle was injected in 4 zones. The inclusion of the sternocleidomastoid muscle was based on its potential role in cervical–mandibular functional interactions and referred pain patterns. In the upper insertion (superior nuchal line of the occiput and mastoid process) 0.2 mL = 10 U, followed by 3 injections of 0.1 mL = 5 U in the superior, middle, and inferior zones. Lastly, the patient received injections inside the mouth, in the anterior border of the masseter muscle. Three areas were injected with 0.1 mL = 5 U each injection. In total, per side the distribution was: (1) masseter muscle: 15 U intra-oral + 25 U extra-oral; (2) temporalis muscle: 35 U; (3) sternocleidomastoid: 25 U. All procedures were carried out under aseptic conditions, with patients in a semi-reclined position. Following the procedure, all patients were instructed to follow a soft diet for three consecutive days to reduce mechanical load on the injected muscles. Additionally, each patient underwent a structured program of five physiotherapy sessions, initiated between 10 and 15 days post-injection. These adjunctive therapies aimed to enhance neuromuscular re-education, promote correct mandibular function, and minimize compensatory habits that could contribute to symptom recurrence. In addition, patients were provided with behavioral counselling to support muscular relaxation and prevent recurrence of parafunctional activities. They were instructed to: avoid clenching or grinding their teeth during the day (diurnal bruxism); use their teeth exclusively for chewing (avoiding nail biting, pen chewing, or holding objects between the teeth); maintain good sleep hygiene, including a minimum of 7 h of sleep per night; avoid the use of screens or digital devices that emit artificial light immediately before bedtime. Starting on the fourth day after injection, patients were advised to apply moist heat to the masticatory muscles for 20 min, followed by gentle self-massage of the treated areas, two to three times daily, to facilitate muscle relaxation and improve blood flow.

### 5.4. Statistical Analysis

All statistical analyses were performed using IBM SPSS Statistics (version 26.0.0; IBM Corp., Armonk, NY, USA) and GraphPad Prism (version 10.4.2; GraphPad Software, San Diego, CA, USA). Descriptive statistics were used to summarize baseline demographic and clinical data. Categorical variables are presented as absolute frequencies and percentages, while continuous variables were expressed as mean ± standard deviation (SD), whereas ordinal or non-normally distributed variables are presented as median and interquartile range (IQR). Normality of continuous variables was assessed using the Shapiro–Wilk test. Comparisons between baseline and 12-month follow-up values were performed using paired statistical tests. The paired Student’s t-test was used for normally distributed continuous variables, while the Wilcoxon signed-rank test was used for ordinal or non-normally distributed variables. Accordingly, myalgia degree was analyzed as an ordinal variable using the Wilcoxon signed-rank test. For continuous paired outcomes, mean differences with 95% confidence intervals (95% CI) were reported. Effect sizes were calculated using Cohen’s d for paired comparisons. For nonparametric paired analyses, effect size was estimated using r, derived from the standardized test statistic. All tests were two-tailed, and a *p*-value < 0.05 was considered statistically significant.

## Figures and Tables

**Table 1:** Baseline characteristics of patients.

Variables	*n* (%) or Mean ± SD (Min–Max)
Number of patients	98
Gender	
Female Male	84 (85.71%)
	14 (14.29%)
Age (years)	45.55 ± 16.27 (18–77)

**Table 2:** Clinical outcomes.

Clinical Diagnosis	Total (*n* = 98)
Myogenous Diagnosis	98 (100%)
Myalgia Degree (0–3)	3.0 (IQR: 2.0–3.0)
Patient-reported orofacial pain intensity (0–10)	2.44 ± 2.54
MMO (mm)	42.55 ± 16.72

**Table 3:** Proportion of patients meeting clinically favorable outcome criteria for each component at baseline and 12-month follow-up. Values are presented as n (%).

Favorable Outcome	Pre-Treatment *n* (%)	Post-Treatment *n* (%)
Myalgia (grade 0–1)	0 (0%)	83 (84.7%)
Patient-reported orofacial pain intensity (VAS ≤ 2)	55 (56.1%)	91 (92.9%)
MMO ≥ 35 mm	95 (96.9%)	97 (99.0%)

**Table 4:** Summary of reintervention outcomes during the 12-month follow-up period after initial incobotulinumtoxinA treatment for myogenous TMD. The table details the number and percentage of patients who underwent reintervention, the types of additional treatments performed, and the clinical classification of symptom recurrence as either myogenous or arthrogenous in origin.

Outcome	N	%
Patients with favorable outcome	78	79.6%
Patients with clinical indication for reintervention	20	20.4%
Myogenous TMD-origin	12	12.2%
Arthrogenous TMD-origin	8	8.2%
Patients who underwent reintervention during follow-up	12	12.2%
Repeat IncobotulinumtoxinA injections	7	7.1%
TMJ arthrocentesis	5	5.1%

**Table 5:** Criteria for classification of favorable outcome.

Criteria	Description
Favorable outcome	No myalgia/pain or only mild myalgia/pain (myalgia degree = 0–1 or pain = 0–2 on a 0–10 VAS scale) and MMO ≥ 35 mm
Treatment failure	Persistent or moderate myalgia/pain (myalgia degree ≥ 2 or pain ≥ 3 on a 0–10 VAS scale) or MMO < 35 mm

## Data Availability

The data presented in this study are available in this article.
